# Socioeconomic status affects achievement of blood pressure target in hypertension: contemporary results from the Swedish primary care cardiovascular database

**DOI:** 10.1080/02813432.2021.2004841

**Published:** 2021-11-24

**Authors:** Georgios Mourtzinis, Karin Manhem, Thomas Kahan, Linus Schiöler, Jetish Isufi, Charlotta Ljungman, Tobias Andersson, Per Hjerpe

**Affiliations:** a Department of Molecular and Clinical Medicine, Institute of Medicine, Sahlgrenska Academy, University of Gothenburg; and Sahlgrenska University Hospital, Gothenburg, Sweden; b Division of Cardiovascular Medicine, Department of Clinical Sciences, Karolinska Institutet, Danderyd Hospital, Stockholm, Sweden; c Occupational and Environmental Medicine, School of Public Health and Community Medicine, Institute of Medicine, Sahlgrenska Academy, University of Gothenburg, Gothenburg, Sweden; d School of Public Health and Community Medicine, Institute of Medicine, Sahlgrenska Academy, University of Gothenburg, Gothenburg, Sweden; e Närhälsan Norrmalm Health Centre, Skövde, Sweden; f R&D Centre Skaraborg Primary Care, Skövde, Sweden

**Keywords:** Hypertension, socioeconomic factors, blood pressure control, primary health care, Sweden

## Abstract

**Objective:**

To assess the relation between socioeconomic status and achievement of target blood pressure in hypertension.

**Design:**

Retrospective longitudinal cohort study between 2001 and 2014.

**Setting:**

Primary health care in Skaraborg, Sweden.

**Subjects:**

48,254 patients all older than 30 years, and 53.3% women, with diagnosed hypertension.

**Main outcome measures:**

Proportion of patients who achieved a blood pressure target <140/90 mmHg in relation to the country of birth, personal disposable income, and educational level.

**Results:**

Patients had a lower likelihood of achieving the blood pressure target if they were born in a Nordic country outside Sweden [risk ratio 0.92; 95% confidence interval (CI) 0.88–0.97], or born in Europe outside the Nordic countries (risk ratio 0.87; 95% CI 0.82–0.92), compared to those born in Sweden. Patients in the lowest income quantile had a lower likelihood to achieve blood pressure target, as compared to the highest quantile (risk ratio 0.93; 95% CI 0.90–0.96). Educational level was not associated with outcome. Women but not men in the lowest income quantile were less likely to achieve the blood pressure target. There was no sex difference in achieved blood pressure target with respect to the country of birth or educational level.

**Conclusion:**

In this real-world population of primary care patients with hypertension in Sweden, being born in a foreign European country and having a lower income were factors associated with poorer blood pressure control.

**KEY POINTS**
The association between socioeconomic status and achieving blood pressure targets in hypertension has been ambiguous.
•In this study of 48,254 patients with hypertension, lower income was associated with a reduced likelihood to achieve blood pressure control.
•Being born in a foreign European country is associated with a lower likelihood to achieve blood pressure control.
•We found no association between educational level and achieved blood pressure control.

## Introduction

Hypertension is a leading and modifiable risk factor for global disease burden [1]. There is solid evidence that antihypertensive drug treatment reduces the risk of cardiovascular disease and death [2]. However, less than half of the treated patients achieve a blood pressure target below 140/90 mmHg, with some regional differences and differences in relation to socioeconomic status within a country or geographic region [3,4]. Low socioeconomic status has been presented as a risk marker of uncontrolled blood pressure, proposing a target for improving antihypertensive treatment [5]. However, studies on possible associations between determinants of socioeconomic status and achieved blood pressure have been ambiguous and vary between populations and regions. Thus, low educational level, but not lack of economic resources, was associated with higher blood pressure in a population in northern Sweden [6]. On the contrary, a recent Japanese study found no clear association between socioeconomic status and blood pressure control [7]. Of note, socioeconomic status is a complex concept depending on a combination of variables [8]. Some of these are more difficult to determine (e.g. wealth) than others (e.g. sex, ethnicity, and educational level).

The current study aims to enlighten the association of attaining blood pressure target achievement and socioeconomic status in a hypertensive population in a Swedish primary care setting. More specifically, the study assessed the association between achieved blood pressure and markers of socioeconomic status (i.e. country of birth, income, and educational level), and the relation to sex.

## Methods

### Study setting: Swedish primary care cardiovascular database

The Swedish Primary Care Cardiovascular Database (SPCCD) is a research project including patients who attended primary care in two Swedish regions (Skaraborg and Stockholm) 2001–2008 with a registered diagnosis of hypertension [9]. A focused update of SPCCD has recently been completed, covering a wider range of patients including also hypertension-related diagnoses, and with an extended period of inclusion and follow-up, but limited to only one region (Skaraborg; SPCCD-SKA). SPCCD-SKA comprises data from ∼75,000 patients 30 years or older attending 20 out of a total of 30 primary care centres 2001–2014 with a registered diagnosis (International Statistical Classification of Diseases-10th Revision, ICD-10) of hypertension I10–15, coronary heart disease I20–25, atrial flutter or atrial fibrillation I48, heart failure I50, cerebrovascular disease I60–69, peripheral artery disease I70–74, diabetes mellitus E10–11 and E14, or chronic renal failure N18. Clinical data were extracted from primary health care electronic medical records by purpose-built software. This included temporal data on age, sex, height, weight, systolic and diastolic blood pressures, smoking status, total cholesterol, low-density, and high-density lipoprotein cholesterol, cystatin C, creatinine, potassium, sodium, uric acid, haemoglobin, glucose, glycated haemoglobin A1c, international normalised ratio of prothrombin complex, microalbuminuria, and diagnoses registered in primary care. The unique Swedish personal identity number was used to link clinical data from each individual patient with information from four national registers. The National Patient Register provides all hospital in-patient and out-patient diagnoses [10]. The Swedish Cause of Death Register provides all mortality data [11]. The Swedish Prescribed Drug Register provides data about all prescribed drugs that are dispensed in Sweden [12]. The Longitudinal integrated database for health insurance and labour market studies managed by Statistics Sweden provides information about the country of birth, level of education, and personal disposable income for the whole population in Sweden [13].

### Study population

This retrospective observational study includes individuals with a diagnosis of hypertension (ICD-10: I10–15) and a documented initial blood pressure value included in SPCCD-SKA between 1 January 2001 and 31 December 2014. Socioeconomic status was assessed at the time of inclusion in SPCCD-SKA (baseline). Definitions for country of birth, educational level, and personal disposable income are given in Table 1. Personal disposable income is an income measurement, which includes all taxable and tax-free income minus taxes and negative transfers. Furthermore, the personal disposable income includes gains/losses arising from a sale (realization) of assets, for example, stocks, mutual funds, or real estate. Baseline comorbidity data (collected from the National Patient Register) were defined as coronary heart disease I20–25, cerebrovascular disease I60–69, heart failure I50, diabetes mellitus E10–11 and E14, atrial flutter or atrial fibrillation I48, depression F31–33, anxiety F41, and psychosis F23 and F29.

**Table 1.** Variables of socioeconomic status.

Country of birth	A. Sweden
	B. Nordic countries except Sweden
	C. Europe except the Nordic countries
	D. Outside Europe
Educational level	A. Primary and secondary education (total <12 years)
	B. Secondary education (total 12 years)
	C. Post-secondary education (total >12 and <15 years)
	D. Post-secondary education (total ≥15 years)
Personal disposable income	Annual quintiles (1st lowest, 5th highest income)

The Nordic countries include Sweden, Norway, Denmark, Finland, and Island. Disposable income is the sum of all taxable and tax-free income minus taxes and negative transfers. The income includes gains/losses arising from a sale (realization) of assets, for example, stocks, mutual funds, or real estate.

### Outcome

The study outcome was defined as achieved blood pressure target <140/90 mmHg, as suggested in the national and international guidelines at the time of the study. According to prevailing national recommendations by the time of the study, blood pressure should be measured in the supine or seated position by a nurse or a physician by an oscillometric device or by the auscultatoric method as an average of two or more recordings. There are currently no home blood pressure measurements included in SPCCD. All patients were followed until 31 December 2014 or death. Achieved blood pressure was defined as the most recent blood pressure registered in SPCCD-SKA.

### Statistics

Data are presented as proportions or as mean values ± standard deviation (*SD*), as appropriate. Multiple logistic regression was used to calculate risk ratios and 95% confidence intervals (CI) for the relationship between socioeconomic variables at baseline and achieved blood pressure target. These analyses were conducted in five different models. In models 1–3 the relationship was evaluated separately for each socioeconomic variable, adjusted for age, sex, and index year. In model 4 all three socioeconomic variables were included in the same analysis adjusted for age, sex, index year, and in addition for marital status and comorbidities (coronary heart disease, cerebrovascular disease, heart failure, diabetes mellitus, atrial flutter/fibrillation, depression, anxiety, and psychosis). In model 5 sex-stratified logistic regression models adjusted for age, index year, marital status, and comorbidities were performed as well. A sensitivity analysis investigated the results in models 1–4 when the blood pressure target was set as ≤140/90 mmHg (instead of <140/90 mmHg) to account for bias due to digit preference. All analyses were conducted using SAS version 9.3 (SAS Institute, Cary, NC, USA).

## Results

We identified 48,254 individuals (53.3% women, 46.7% men), and the mean follow-up time was 6 years. Half of the individuals had the last blood pressure measurement in 2014. Baseline characteristics are presented in Table 2. Men were younger, had more comorbidities, and lower systolic and higher diastolic blood pressures. In addition, men, in general, achieved higher educational levels, and higher incomes, as compared to women.

**Table 2.** Baseline characteristics.

	All	Men	Women
*n* (%)	48,254	22,526 (46.7)	25,728 (53.3)
Age, years (mean ±*SD*)	66 ± 13	65 ± 12	67 ± 13
Systolic blood pressure, mm Hg (mean ±*SD*)	156.7 ± 17	154.5 ± 22	158.5 ± 22
Diastolic blood pressure, mm Hg (mean ±*SD*)	86.2 ± 11	87.1 ± 13	85.5 ± 12
Total cholesterol, mmol/L (mean ±*SD*)	5.39 ± 1.07	5.18 ± 1.04	5.60 ± 1.05
Current or former tobacco smoking (%)	15,789 (32.7)	9351 (37)	7054 (25)
Coronary heart disease (%)	4903 (10.2)	3031 (13.5)	1872 (7.3)
Cerebrovascular disease (%)	3552 (7.4)	1933 (8.6)	1619 (6.3)
Heart failure (%)	1433 (3.0)	764 (3.4)	669 (2.6)
Diabetes mellitus (%)	2987 (6.2)	1714 (7.6)	1273 (4.9)
Atrial flutter and/or atrial fibrillation (%)	2469 (5.1)	1350 (6.0)	1119 (4.3)
Depression (%)	760 (1.6)	332 (1.5)	428 (1.7)
Anxiety (%)	445 (0.9)	203 (0.9)	242 (0.9)
Psychosis (%)	71 (0.1)	41 (0.2)	30 (0.1)
Country of birth			
Sweden (%)	43,979 (91.2)	20,601 (91.5)	23,378 (90.9)
Nordic countries except Sweden (%)	2174 (4.5)	951 (4.2)	1223 (4.8)
Europe except the Nordic countries (%)	1410 (2.9)	641 (2.8)	769 (3.0)
Outside Europe (%)	691 (1.4)	333 (1.5)	358 (1.4)
Income			
1st quantile (lowest income)	10,111(21.0)	2230 (9.9)	7882 (30.8)
2nd quantile	9551 (19.9)	3478 (15.3)	6073 (23.7)
3rd quantile	9362 (19.5)	4670 (20.8)	4692 (18.2)
4th quantile	9619 (20.0)	5230 (23.3)	4389 (17.1)
5th quantile (highest income)	9400 (19.6)	6816 (30.4)	2584 (10.1)
Education level			
Primary and secondary education, total <12 years (%)	36,840 (76.3)	16,529 (73.4)	20,311 (78.9)
Secondary education, total 12 years (%)	3956 (8.2)	2521 (11.2)	1435 (5.6)
Post-secondary education, total >12 and <15 years (%)	3197 (6.6)	1561 (7.1)	1636 (6.4)
Post-secondary education, total ≥15 years (%)	3451 (7.2)	1588 (7.1)	1863 (7.2)
Marital status			
Single/divorced (%)	12,826 (26.6)	6910 (30.7)	5916 (23.0)
Married (%)	27,235 (56.5)	14,083 (62.6)	13,152 (51.2)
Widowed (%)	8129 (16.9)	1503 (6.7)	6626 (25.8)

SD: standard deviation.

Data are numbers, unless otherwise indicated. The Nordic countries includes Sweden, Norway, Denmark, Finland, Iceland. Missing values for educational level 810 (1.7%), smoking habits 11,219 (23%), and total cholesterol 13,952 (29%).

At follow-up 24,114 individuals (50.0%) achieved blood pressure target; 12,306 (47.8%) of the women, and 11,808 (52.4%) of the men. Three logistic regression models (adjusted for age, sex, and index year) were used to estimate the risk ratio for achieved blood pressure target, in relation to indices of socioeconomic status (Table 3). First, patients had lower likelihoods to achieve the blood pressure target if they were born in another Nordic country than Sweden (risk ratio 0.93; 95% CI 0.89–0.97), or in Europe outside the Nordic countries (risk ratio 0.88; 95% CI 0.83–0.93), as compared to being born in Sweden. Second, income in the lowest quantile was associated with a lower likelihood to achieve blood pressure target, as compared to the highest income quantile (risk ratio 0.94; 95% CI 0.91–0.97). Third, educational level was not associated with achieved blood pressure target.

**Table 3.** Risk ratios for the association of achieved target blood pressure (<140/90 mmHg) with country of birth (A), income (B), and educational level (C) adjusted for age, sex, and index-year.

	Risk ratios (95% CI)	
A. Country of birth		
Nordic countries	0.93	(0.89, 0.97)
Outside Europe	0.98	(0.92, 1.06)
Europe except Nordic countries	0.88	(0.83, 0.93)
Sweden	Reference	
B. Personal disposable income		
Income 1st quantile (lowest income)	0.94	(0.91, 0.97)
Income 2nd quantile	0.97	(0.94, 1.00)
Income 3rd quantile	1.01	(0.99, 1.04)
Income 4th quantile	1.02	(0.99, 1.05)
Income 5th quantile (highest income)	Reference	
C. Educational level		
Education <12 years	0.99	(0.96, 1.03)
Education 12 years	1.01	(0.97, 1.06)
Education >12 and <15 years	1.01	(0.98, 1.06)
Education ≥15 years	Reference	

CI: confidence interval.

The Nordic countries includes Sweden, Norway, Denmark, Finland, Iceland. Income has been further adjusted for country of birth and educational level. Educational level has been further adjusted for country of birth.

The fourth logistic regression model, including all socioeconomic variables, was conducted with additional adjustment for marital status and history of comorbidity. The relations are presented in Table 4. Again, educational level was not associated with achieved blood pressure target. Women (but not men) in the lowest income quantile, had a lower likelihood to achieve blood pressure target. There was no sex difference in the achieved blood pressure target with respect to the country of birth or educational level. The adjusted logistic regression model conducted for women and men separately showed similar results as for the entire study population (Table 5).

**Table 4.** Risk ratios for blood pressure target (<140/90 mmHg) achievement adjusted for age, sex, index-year, country of birth, income, education, marital status, and comorbidities.

	Risk ratios (95% CI)	
Nordic countries	0.92	(0.88, 0.97)
Outside Europe	0.97	(0.91, 1.04)
Europe except Nordic countries	0.87	(0.82, 0.92)
Sweden	Reference	
Income 1st quantile (lowest income)	0.93	(0.90, 0.96)
Income 2nd quantile	0.97	(0.93, 1.00)
Income 3rd quantile	1.01	(0.98, 1.04)
Income 4th quantile	1.01	(0.99, 1.04)
Income 5th quantile (highest income)	Reference	
Education <12 years	0.99	(0.96, 1.03)
Education 12 years	1.01	(0.97, 1.06)
Education >12 and <15 years	1.01	(0.97, 1.16)
Education ≥15 years	Reference	

CI: confidence interval.

The Nordic countries includes Sweden, Norway, Denmark, Finland, Iceland. Comorbidities included in the analyses are coronary heart disease, cerebrovascular disease, heart failure, diabetes mellitus, atrial fibrillation/flutter, depression, anxiety, and psychosis.

**Table 5.** Risk ratios for blood pressure target (<140/90 mmHg) achievement in men and women, adjusted for age, sex, index-year, country of birth, income, education, marital status, and comorbidities.

	Men		Women	
	Risk ratios (95% CI)		Risk ratios (95% CI)	
Nordic countries	0.93	(0.87, 0.99)	0.92	(0.86, 0.98)
Outside Europe	1.01	(0.92, 1.11)	0.94	(0.85, 1.04)
Europe except Nordic countries	0.84	(0.77, 0.91)	0.90	(0.84, 0.97)
Sweden	Reference		Reference	
Income 1st quantile (lowest income)	0.96	(0.91, 1.00)	0.93	(0.88, 0.98)
Income 2nd quantile	0.94	(0.90, 0.99)	0.97	(0.92, 1.02)
Income 3rd quantile	0.99	(0.096, 1.03)	0.98	(0.94, 1.03)
Income 4th quantile	0.99	(0.96, 1.03)	1.00	(0.96, 1.05)
Income 5th quantile (highest income)	Reference		Reference	
Education <12 years	0.98	(0.94, 1.03)	1.03	(0.98, 1.08)
Education 12 years	1.00	(0.94, 1.06)	1.02	(0.96, 1.09)
Education >12 and <15 years	0.99	(0.93, 1.06)	1.04	(0.97, 1.10)
Education ≥15 years	Reference		Reference	

CI: confidence interval.

The Nordic countries includes Sweden, Norway, Denmark, Finland, Iceland. Comorbidities included in the analyses are coronary heart disease, cerebrovascular disease, heart failure, diabetes mellitus, atrial fibrillation/flutter, depression, anxiety, and psychosis.

A sensitivity analysis with ≤140/90 as blood pressure target showed that 63.6% of the patients reached target blood pressure. The association of country of birth and educational level to achieved blood pressure remained the same. However, the effect of the income was attenuated and no longer significant with a blood pressure target set to ≤140/90 mmHg.

## Discussion

### Principal findings

There are three major findings of this observational study among almost 50,000 patients with hypertension in Swedish primary care. First, lower disposable income was associated with a lower likelihood of achieving the blood pressure target. Second, individuals born in a European country other than Sweden had a lower likelihood of achieving the blood pressure target. Third, educational level was not associated with achieving the blood pressure target.

### Strengths and weaknesses of the study

The large size of this contemporary real-world study population, the longitudinal design, and the robust data of the conditions for exposure (i.e. several socioeconomic variables) are strengths of the present study. However, some important limitations need to be acknowledged. First, patients were treated according to standard clinical practice, which may result in treatment bias by indication. Second, the observational retrospective study design is sensitive for confounding. We attempted to adjust for potential confounders by multiple analyses to reduce the risk of confounding. We have included cardiovascular comorbidity and diabetes mellitus in the adjustments since those diagnoses result in a more structured follow-up that can affect achieved blood pressure. However, data about diet, alcohol consumption, exercise habits, and other possible factors affecting blood pressure were not available to us, a common challenge in large observational studies. Third, the personal disposable income might misclassify patients with low personal income who live in a rich households. Therefore, we have adjusted our analyses for marital status. Finally, the results may not be valid in other contexts, where different conditions may prevail.

### Relation to other studies

The main finding of this study is the independent association between low personal disposable income and lower likelihood to achieve blood pressure target. This is in agreement with our previous findings, where patients with higher income had lower discontinuation rates of antihypertensive drug treatment, while there was no association between educational level and treatment persistence [14]. This may, at least in part explain our findings. Previous studies have presented conflicting results regarding the relations between income and blood pressure control. However, the impact of income depends partly on social infrastructure, which varies between different societies and countries. Perhaps more important, different methods to measure economic disparities have been used in previous studies, including simple questions, self-administered questionnaires [7,15], and comparisons between economic status in different areas, communities, or countries [16]. We stratified all individuals according to their personal disposable income acquired directly from a validated national register. Moreover, we have previously shown that low income is associated with a higher risk of mortality and cardiovascular events in patients with hypertension [17]. Our results suggest that patients with low income should be offered increased attention to ascertain adequate blood pressure control to prevent hypertensive end-organ damage and subsequent cardiovascular morbidity and mortality.

In this study, hypertensive patients born in a European country outside Sweden were less likely to achieve blood pressure targets. Being a foreigner might be an obstacle in establishing good contact with the healthcare system, although Sweden provides equal subsidised accessible universal healthcare for all citizens. Previous studies have shown differences in the prevalence of hypertension among immigrants [18], and ethnical differences in blood pressure control [19,20]. However, no consistent difference in blood pressure control was found between any of the European nationalities in an investigation performed in Switzerland [21]. Also, we have shown that among hypertensive patients in Sweden, being born in Finland was associated with a higher risk of mortality, while other immigrants had a mortality advantage [22]. Of note, only <8% of the study population in the current study was born in a European country other than Sweden and <2% were born outside of Europe. This calls for caution when interpreting our results regarding the country of birth. Taken together, our results are in line with the five studies mentioned above, suggesting that some immigrants with hypertension need higher attention to achieve blood pressure targets.

We found no association between educational level and blood pressure control. Low socioeconomic status (whether assessed by income, educational level, or occupation) has been associated with an increased prevalence of hypertension [23]. However, the association between educational level and achieved blood pressure target in hypertension remains uncertain. Our findings are in agreement with findings reported from the Nordic countries [24], Japan [7], and Kazakhstan [25], while studies from the United States [15], Romania [26], and China [27] have shown positive correlations between educational level and blood pressure control. Differences in social structure, educational systems, costs for attending higher education, health care organization, and economic systems between countries may in part explain these different results. The effect of educational level on blood pressure achievement is partly mediated by income. However, we did not find an association between educational level and blood pressure control neither before nor after adjustment for personal disposable income.

Up to now, education has been looked upon as a more reliable entity while income may vary during a lifetime. The organisation, or change in organisation, of the educational system, may, however, be of major concern when the association between educational length and health is evaluated. In Sweden, such a change has been implemented during the last 30 years. Earlier, an education length of 12 years was almost identical to a later university degree. Today education of 12 years may include craft training to achieve a vocational degree. This change in the content in the educational system may partly explain why income and not educational length are closely related to blood pressure control. Thus, whereas income, educational level, and occupation are all established markers for socioeconomic status, our findings suggest that personal disposable income has a stronger association than educational level to achieve blood pressure control in a hypertensive population in Sweden.

This study shows that one-half of people attending Swedish primary care in 2001–2014 with a diagnosis of hypertension achieved a blood pressure target of <140/90 mm Hg. This is an encouraging improvement from our previous findings (25% in 2001–2002, and 37% in 2007–2008) [28]. Notwithstanding that the proportion of patients with controlled blood pressure is comparable to findings elsewhere [29], there is room for further improvement. In this study, women were less likely to achieve blood pressure targets. However, there was no significant difference between men and women regarding achieved blood pressure after adjusting for marital status and comorbidity. This is in line with our previous findings, where women obtained a controlled blood pressure less often than men [30]. Women were also offered different antihypertensive drug therapy, more often diuretics or beta-blockers, and less often angiotensin-converting enzyme inhibitors, as compared to men [30,31]. Other studies have shown better blood pressure control in women than in men, but beyond the age of 60 years, control rates seem to be higher in men [32,33]. Moreover, men have shown to be more persistent in antihypertensive drug therapy than women in an Italian study [34], while male sex was found to be associated with lower persistency to antihypertensive drug treatment in Sweden [14]. The current results extend these findings and relate them to socioeconomic status, where higher income relates to improved blood pressure control in women.

### Meaning of the study

This real-world population study of patients with hypertension attending primary care in Sweden shows that socioeconomic status, i.e. low income, and country of birth in a foreign European country, was associated with inferior blood pressure control in both women and men. Thus, health care providers are advised to provide additional support for those patients to achieve proposed blood pressure targets and thereby reduce future cardiovascular risk.
